# Understanding the neuroprotective effect of tranexamic acid: an exploratory analysis of the CRASH-3 randomised trial

**DOI:** 10.1186/s13054-020-03243-4

**Published:** 2020-11-11

**Authors:** Amy Brenner, Antonio Belli, Rizwana Chaudhri, Timothy Coats, Lauren Frimley, Sabariah Faizah Jamaluddin, Rashid Jooma, Raoul Mansukhani, Peter Sandercock, Haleema Shakur-Still, Temitayo Shokunbi, Ian Roberts, On behalf of the CRASH-3 trial collaborators, Ian Roberts, Haleema Shakur-Still, Amy Aeron-Thomas, Antonio Belli, Amy Brenner, Muhammad Anwar Chaudary, Rizwana Chaudhri, Sabariah Faizah Bt Jamaluddin, Lauren Frimley, Kiran Javaid, Rashid Jooma, Aasia Kayani, Caroline Leech, Khalid Mahmood, Raoul Mansukhani, Julina Md Noor, Jorge Mejia-Mantilla, Phil Moss, Jason Pott, Peter Sandercock, Temitayo Shokunbi, Liliana Vallecilla, Henry Benjamin Hartzenberg, Manjul Joshipura, Pablo Perel, Michael J. Clarke, Samuel C. Ohaegbulam, Anthony Rodgers, Tony Brady, Yashbir Dewan, Phil Edwards, Edward O. Komolafe, Monica Arribas, Emma Austin, Eni Balogun, Lin Barneston, Collette Barrow, Danielle Beaumont, Myriam Benyahia, Imogen Brooks, Madeleine Cargill, Laura Carrington, Lisa Cook, Beatrice Cornu-Hewitt, Amber Geer, Daniel Gilbert, Catherine Gilliam, Julio Gil-Onandia, Daniel Hetherington, Courtenay Howe, Carolyn Hughes, David I’anson, Rob Jackson, Miland Joshi, Sneha Kansagra, Taemi Kawahara, Katharine Ker, Sergey Kostrov, Abda Mahmood, Hakim Miah, Bernard Ndungu, Kelly Needham, Cecilia Okusi, Aroudra Outtandy, Raul Pardinaz-Solis, Daniel Pearson, Tracey Pepple, Claude Pisani, David Prieto-Merino, Danielle Prowse, Nigel Quashi, Anna Quinn, Maria Ramos, Mia Reid, Chris Roukas, Giulia Scrapa, Chelci Squires, Jemma Tanner, Andrew Thayne, Lesley Vidaurre, Elizabeth Woods, Bukola Fawole, Olusade Adetayo, Olujide Okunade, Tamar Gogichaishvili, Maria de los Angeles Munoz-Sanchez, Fatos Olldashi, Satish Krishnan, Vincent Djientcheu, Jorge Loria Castellanos, Frank Rasulo, Qadamkhear Hama, Yakub Mulla, Ioan Stefan Florian, Juan Tobar, Hussein Khamis, Conor Deasy, Bobby Wellsh, Jean Williams-Johnson, Susilo Chandra, Vincent Mutiso, Rizwan Butt, Muhammad Hammad Nasir, Salman Ahmad, Farwah Aslam, Khurram Ishaque, Faheem Usmani, Shahrukh Rizvi, Farhad Ali, Omair Sajjad, Ali Zunair, Lal Rehman, Raza Rizvi, Farrukh Javeed, Shakeel Ahmed, Asad Abbas, Ali Afzal, Ali Mikdad, Asif Bashir, Anwar Chaudary, Tariq Salahuddin, Bashir Ahemed, Amir Aziz, Naveed Ashraf, Shahzad Hussain, Usman Ahmad, Muhammad Asif, Muhammad Adil, Adeel Rauf, Rizwan Khan, Bilal Ahmad, Umair Afzal, Hassan Raza, Quratul Ain, Sajjad Yaqoob, Qaiser Waseem, Muffasser Nishat, Suneel Semvel, Javed Iqbal, Samra Majeed, Sana Zulfiqar, Madeeha Iqbal, Nazia Majeed, Manzoor Ahmed, Nadeem Akhtar, Mohammad Malik, Yasir Shehzad, Muhammad Yousaf, Abdul Wahid, Abdul Samad, Saifullah Shah, Mumtaz Ali, Jehan Zeb, Abdus Salam Khan, Adeela Irfan, Salman Sharif, Riaz Memon, Ben Bloom, Tim Harris, Imogen Skene, Geoffrey Bellhouse, Olivia Boulton, Geraldine Ward, Catherine Jarvis, Carly Swann, Sathananathan Ratnam, Ronald Carrera, Kamal Yakoub, David Davies, Emma Fellows, Heather Jarman, Sarah Rounding, Elizabeth Johnson, Catherine Loughran, Fiona Lecky, Kate Clayton, Angiy Michael, Angela Coumbarides, Jason Kendall, Beverley Faulkner, Ruth Worner, Emma Gendall, Philip Hopkins, Paul Riozzi, Hannah Cotton, Raine Astin-Chamberlain, Mark Wilson, Jan Bodnar, Rachel Williams, Alberto Rigoni, Abdo Sattout, John Fletcher, Calum Edge, Nina Maryanji, Adrian Boyle, Susie Hardwick, Ellen Nichols, Catherine Hayhurst, Frank Coffey, Chris Gough, Philip Miller, Lucy Ryan, Melanie Darwent, Alexis Espinosa, Sally Beer, Julie Norton, Holly Maguire, Kay Finney, Anthony Kehoe, Rosalyn Squire, Alison Jeffery, Christiane Vorwerk, Denise Foord, Eliot Wilkinson, Avril Kuhrt, Shammi Ramlakhan, Stuart Reid, Andy Curran, Sean McMullan, Tajek Hassan, Stuart Nuttall, Stephen Haig, Saif Al-Nahhas, Diederik Bulters, Ardalan Zolnourian, Tamsin Ribbons, Ian Mew, Tanya de Weymarn, Victoria Hughes, Jane McVicar, Cieran McKiernan, Liza Keating, Henrik Reschreiter, Judith Wright, Louisa Chan, Himanshu Kataria, Alastair Ireland, Richard Body, Alasdair Corfield, Shindo Francis, William Townend, Timothy Coats, James Gagg, Sarah Wilson, Rowley Cottingham, Simon Tucker, Frank Sutherland, Louisa Mitchell, Lucy Parker, Ola Afolabi, Fiona Hunter, Mark Jadav, Kayode Adeboye, Mandy Grocutt, Gabrielle May, David Watson, Andrea Wootten, Sarah Robertshaw, Susan Dorrian, Rob Perry, Hyun Choi, Claire McGroarty, Paul Shone, David Maritz, Sabariah Jamaluddin, Julina Noor, Norizan Rosli, Leonard Leong Sang Xian, Yong De Jun, Fatahul Mohamed, Cheng Hee Song, Arman Hawari, Leong Yuen Chin, Hardawani Mohd Hussein, Mohd Lotfi, Hafiq Hamid, Nujaimin Udin, Peck Lian, See Choo, Kwanhathai Wong, Fathiyah Gani, Mardhiah Jusoh, Darrsini Rajakumar, Chia Boon Yang, Nur Shahidah Binti Dzulkiflee, Wong Chok Ky, Muhaimin Azwan Bin Mohd Azman, Adi Bin Osman, Azma Haryaty Ahmad, Ramzuzaman Ismail, Si Qi Lai, Mohd Amin Bin Mohidin, Norwani Binti Deraman, Salliza Binti Selamat, Ida Abidin, Nurkhairulnizam Halim, Zuraini Bakar, Zainalabidin Mohamed Ismail, Badrul Hisham, Ruhaida Kamal, Zainal Effendy, Mashitah Ismail, Noor Azleen, Liu Yeo Seng, Kamarul Aryffin Baharuddin, Regunath Kandasamy, Azlan Kamalludin, Shamsul Asmee, Mohd Fadzil, Ahmad Basitz, Norhaya Abdullah, Giorgi Ingorokva, Shota Ingorokva, Iamze Agdgomelashvili, Kote Mumladze, Ioseb Maisuradze, Iulia Kugusheva, Buba Shalamberidze, Gia Tomadze, Juan Fernandez-Ortega, Raimundo Seara-Valero, Guillermo Ibañez-Botella, Victoria Garcia-Martinez, Melida Garcia Martul, Santiago Freita Ramos, Guillermo Lago Preciado, Claudio Garcia-Alfaro, Angeles Munoz-Sanchez, Rafael Bellido-Alba, Carmen Corcobado, Ana Bueno, Alfonso Ambros, Juan Tihista Jimenez, Jose Roldan Ramirez, José Martín, Laura Inés Rodríguez, Jaime Fontanals, José Manuel Jiménez-Moragas, Joaquín Paya Berbegal, Olaomi Oluwole, Raji Mahmud, Nancy Ukwu, Femi Bankole, Abidemi Oseni, Bamidele Adebayo, Adefolarin Malomo, Liadi Tiamiyu, Adefisayo Adekanmbi, Lateef Thanni, Ayodeji Olubodun, Fidelis Ojeblenu, Michael Uwaezuoke, Edward Komolafe, Oluwafemi Owagbemi, Fatai Ishola, Adewumi Durodola, Ukpong Udoffa, Adeniran James, Azeez Tella, Andrew Dongo, Uchechi Ekpemiro, Stanley Anyanwu, Nafiu Aigoro, Wilfred Mezue, Danaan Shilong, Abiodun Azeez, Olakunle Babalola, Mohammed Ibrahim, Joseph Obande, Alfredo Constain Franco, Edwin Vasquez Salazar, Sebastian Betancur Londoño, Viviana Medina Cardona, Carlos Morales, Santiago Naranjo, July Agudelo, Sandra Carvajal, Yidhira Fajardo-Gaviria, Yam Roka, Ushma Ghising, Narayani Roka, Manzil Shrestha, Upendra Devkota, Bivek Vaidya, Pankaj Nepal, Amit Thapa, Bidur KC, Ajit Shrestha, Rajiv Jha, Prabin Shrestha, Irgen Hodaj, Erion Spaho, Asllan Selaj, Nirian Bendo, Tomohisa Shoko, Hideki Endo, Atsushi Senda, Yasushi Hagihara, Takashi Fuse, Naohisa Masunaga, Yasuhiro Otomo, Ryuichiro Egashira, Takahiro Ohnuki, Alya AlMazmi, Subrata Saha, Alexander Suvarov, Than Latt Aung, Kaung Myat Tun, Tint Tint Khaing, Thinzar Maw, Orlane Ndome, Mireille Moumi, André Mbida, Joseph Fondop, Mba Sebastien, Abdul Azim, Jan Adil, Zabiullah Amiry, Jorge Loría-Castellanos, Nancy Guevara Rubio, Patricia Ortega Leon, Francisco Estrada, Erandy Montes de Oca-García, Hafid Sanchez, Angélica Soria, Paola Bonucci, Federico Franchi, Alan Girardini, Himdad Hameed, Muhammad Basim, Simon Stock, Eap Hourt, Ali Ilunga, Jonathan Mulenga, Horia Ples, Adam Danil, Mircea Gorgan, Ioan Florian, Dusan Vlahovic, James French, Jeffrey East, Antonius Kurniawan, Julius Kiboi

**Affiliations:** 1 London School of Hygiene & Tropical Medicine, Keppel Street, London, WC1E 7HT UK; 2 College of Medical and Dental Sciences, University of Birmingham, Birmingham, UK; 3 Global Institute of Human Development, Shifa Tameer-e-Millat University, Rawalpindi, Pakistan; 4 Department of Cardiovascular Sciences, University of Leicester, University Road, Leicester, LE1 7RH UK; 5 Department of Emergency Medicine, Faculty of Medicine, Universiti Teknologi MARA, Sungai Buloh Campus, Shah Alam, Malaysia; 6 Department of Surgery, Aga Khan University Hospital, Karachi, 74800 Pakistan; 7 Centre for Clinical Brain Sciences, University of Edinburgh, Edinburgh, EH16 4SB UK; 8 University College Hospital, Ibadan, Nigeria; 9 Department of Neurological Surgery, PMB 5116, Ibadan, Oyo State Nigeria

**Keywords:** Traumatic brain injury, Tranexamic acid, CRASH-3 trial, Randomised controlled trial, Intracranial haemorrhage, Epidemiology, Emergence care

## Abstract

**Background:**

The CRASH-3 trial hypothesised that timely tranexamic acid (TXA) treatment might reduce deaths from intracranial bleeding after traumatic brain injury (TBI). To explore the mechanism of action of TXA in TBI, we examined the timing of its effect on death.

**Methods:**

The CRASH-3 trial randomised 9202 patients within 3 h of injury with a GCS score ≤ 12 or intracranial bleeding on CT scan and no significant extracranial bleeding to receive TXA or placebo. We conducted an exploratory analysis of the effects of TXA on all-cause mortality within 24 h of injury and within 28 days, excluding patients with a GCS score of 3 or bilateral unreactive pupils, stratified by severity and country income. We pool data from the CRASH-2 and CRASH-3 trials in a one-step fixed effects individual patient data meta-analysis.

**Results:**

There were 7637 patients for analysis after excluding patients with a GCS score of 3 or bilateral unreactive pupils. Of 1112 deaths, 23.3% were within 24 h of injury (early deaths). The risk of early death was reduced with TXA (112 (2.9%) TXA group vs 147 (3.9%) placebo group; risk ratio [RR] RR 0.74, 95% CI 0.58–0.94). There was no evidence of heterogeneity by severity (*p* = 0.64) or country income (*p* = 0.68). The risk of death beyond 24 h of injury was similar in the TXA and placebo groups (432 (11.5%) TXA group vs 421 (11.7%) placebo group; RR 0.98, 95% CI 0.69–1.12). The risk of death at 28 days was 14.0% in the TXA group versus 15.1% in the placebo group (544 vs 568 events; RR 0.93, 95% CI 0.83–1.03). When the CRASH-2 and CRASH-3 trial data were pooled, TXA reduced early death (RR 0.78, 95% CI 0.70–0.87) and death within 28 days (RR 0.88, 95% CI 0.82–0.94).

**Conclusions:**

Tranexamic acid reduces early deaths in non-moribund TBI patients regardless of TBI severity or country income. The effect of tranexamic acid in patients with isolated TBI is similar to that in polytrauma. Treatment is safe and even severely injured patients appear to benefit when treated soon after injury.

**Trial registration:**

ISRCTN15088122, registered on 19 July 2011; NCT01402882, registered on 26 July 2011.

## Background

The acute management of traumatic brain injury (TBI) aims to avoid secondary brain damage and optimise conditions for recovery [1]. The day of the injury is the most hazardous, accounting for one third of in-hospital deaths [2]. Some TBI victims have brain damage that is incompatible with life and die shortly after admission. In many patients, intracranial bleeding starts soon after impact and continues for several hours, with the majority of haematoma expansion occurring within 1–1.5 h of injury [3, 4]. The accumulating blood can increase intracranial pressure, causing cerebral herniation and death. Tranexamic acid reduces bleeding in surgery and reduces death from bleeding in traumatic and post-partum haemorrhage [5–7]. The therapeutic premise of the CRASH-3 trial was that timely tranexamic acid treatment might curtail intracranial bleeding and prevent some of the early bleeding-related deaths. A 1-g bolus started within 3 h of injury was followed by an infusion of 1 g over 8 h. Tranexamic acid has a half-life of 2 h and by the second day is almost completely eliminated. By this time, the bleeding will have stopped, but other pathological processes, likely unaffected by tranexamic acid, will continue to cause deaths. Those who survive the first day run the risk of cerebral oedema, diffuse axonal injury, organ failure, sepsis, pneumonia and many other threats, some iatrogenic, that make up the remaining two thirds of in-hospital deaths.

The management of TBI is only partly based on results from randomised trials. In practice, doctors draw on pathophysiological knowledge, the available evidence and their clinical experience to identify mechanisms of brain damage and target physiologically based treatment accordingly [3]. Large randomised trials can reduce our therapeutic uncertainty, but to categorise them as positive or negative based on arbitrary *p* value thresholds is inappropriate [8–10]. The CRASH-3 trial results have variously been described as ‘negative’, ‘neutral’ or ‘a win for patients with head injury’ that will benefit patients [11–13]. We argue that randomised trials can deepen our understanding of pathophysiology and that mechanistic insights should inform their interpretation. To explore the mechanism of action of tranexamic acid in TBI patients, we examined the timing of its effect on death. We also set the results of our analysis in the context of other trials of tranexamic acid in TBI and polytrauma patients, taking into consideration current treatment guidelines that exclude patients with isolated TBI.

## Methods

The background to the CRASH-3 trial, the methods, baseline characteristics and main results were previously reported [2, 6, 14]. Briefly, adults with TBI who were within 3 h of injury and had a Glasgow coma scale score (GCS) ≤ 12 or any intracranial bleeding on CT scan and no significant extra-cranial bleeding were eligible. The time window for eligibility was originally 8 h, but in 2016, the protocol was changed to limit recruitment to within 3 h of injury. Between July 2012 and January 2019, we randomly allocated 12,737 patients with TBI to receive tranexamic acid or placebo, of whom 9202 patients were treated within 3 h. Patients were assigned by selecting a numbered treatment pack from a box containing eight packs that were identical apart from the pack number. Patients, care givers and those assessing outcomes were masked to treatment allocation.

Based on previous research on the mechanism of tranexamic acid in bleeding trauma patients, we hypothesised that tranexamic acid would have a greater effect on deaths soon after injury, since early bleeding-related deaths have the most potential to be reduced by tranexamic acid [15]. We pre-specified this hypothesis in the statistical analysis plan that we published before un-blinding [14]. We also anticipated that the treatment effect would be diluted by the inclusion of patients with a GCS score of 3 or unreactive pupils who have a very poor prognosis regardless of treatment [14]. The trial results were consistent with both of these hypotheses [2]. The pre-specified primary outcome in the CRASH-3 trial was death due to head injury within 28 days among patients treated within 3 h of injury. Although our scientific reasons for pre-specifying head injury death as the primary outcome were given in the statistical analysis plan and presented in detail elsewhere [16], there has been strong interest in the effects of tranexamic acid on all-cause mortality. As such, this analysis focusses on early deaths from any cause, excluding patients with a GCS score of 3 or bilateral unreactive pupils. Analyses of head injury deaths and analyses including patients with a GCS score of 3 or bilateral unreactive pupils are presented in the Additional file 1 for comparison with the results presented below and for cross-reference with the main trial results.

We examine the temporal distribution of deaths from any cause in the CRASH-3 trial. We explore the effects of tranexamic acid on deaths due to any cause within 24 h of injury and on deaths due to any cause within 28 days, stratified by severity and country income level. We use the baseline GCS score to define severity—mild to moderate (GCS 9–15) and severe (GCS 3–8)—and World Bank definitions to determine country income level (LMIC vs HIC). Because a subgroup analysis demonstrated effect modification by severity, we explore this further. Because most patients were from LMICs, the generalisability of the results to HICs has been questioned and so we explore how the treatment effects vary by country income level. To check if the effect on early deaths could be explained by undiagnosed extra-cranial bleeding, we conducted a sensitivity analysis excluding patients with hypotension (SBP < 90 mmHg). We also examined the effects of tranexamic acid on vascular occlusive events (fatal and non-fatal) in all patients irrespective of time to treatment because theoretically the potential risk of vascular occlusive events would be greater with late treatment as there is a shift from a fibrinolytic to a coagulopathic state. We report risk ratios, 95% confidence intervals and heterogeneity *p* values. We excluded 98 patients with missing outcome data.

We prespecified an analysis setting the results of the CRASH-3 trial in the context of other evidence, including the CRASH-2 trial, in which 40% of deaths were due to head injury [14]. The CRASH-3 trial essentially represents a subgroup of patients with isolated TBI who were excluded from the CRASH-2 trial. Here, to set our results in the context of tranexamic acid in polytrauma patients, we pooled the data from the CRASH-2 and CRASH-3 trials in a one-step fixed effects individual patient data meta-analysis using a Poisson regression model with sandwich variance estimation, adjusted for time to treatment. In the main CRASH-3 trial publication, we updated a systematic search for randomised trials of tranexamic acid in TBI. We searched PubMed, Science Citation Index, National Research Register, Zetoc, SIGLE, Global Health, LILACS, Current Controlled Trials, the Cochrane Injuries Group Specialised Register, CENTRAL, MEDLINE and EMBASE. We identified three trials in addition to the CRASH-3 trial including the CRASH-2 intracranial bleeding study, a randomised trial of 283 TBI patients sponsored by Khon Kaen University [17] and a randomised trial of pre-hospital tranexamic acid in 967 TBI patients sponsored by the University of Washington (NCT01990768). The CRASH-2 intracranial bleeding study was omitted as this is already contained within the CRASH-2 trial dataset, and the small Thai study was omitted due to a lack of data on timing of death, cause of death and GCS score, and limitations in methodological quality including an unclear risk of selection bias from allocation concealment.

The model for the one-step meta-analysis was as follows:

$$ \log\ \pi ={\beta}_0+{\beta}_1\mathrm{trial}+{\beta}_2\mathrm{group}+{\beta}_3\mathrm{ttt} $$

where trial = 0 for CRASH-2 and 1 for CRASH-3, group = 0 for placebo and 1 for TXA, ttt is time to treatment and *β*_2_ is the summary effect estimate across both trials.

We also consider the CRASH-3 trial results in the context of the CRASH-2 trial and the trial of pre-hospital tranexamic acid (NCT01990768) using an aggregate data meta-analysis with fixed effects to assess the effect of tranexamic acid on death at 28 days excluding patients with a GCS score of 3 or bilateral unreactive pupils, and on vascular occlusive events in all patients. An aggregate data meta-analysis was used because we did not have access to the individual patient data for trial NCT01990768.

## Results

**Fig. 1:**
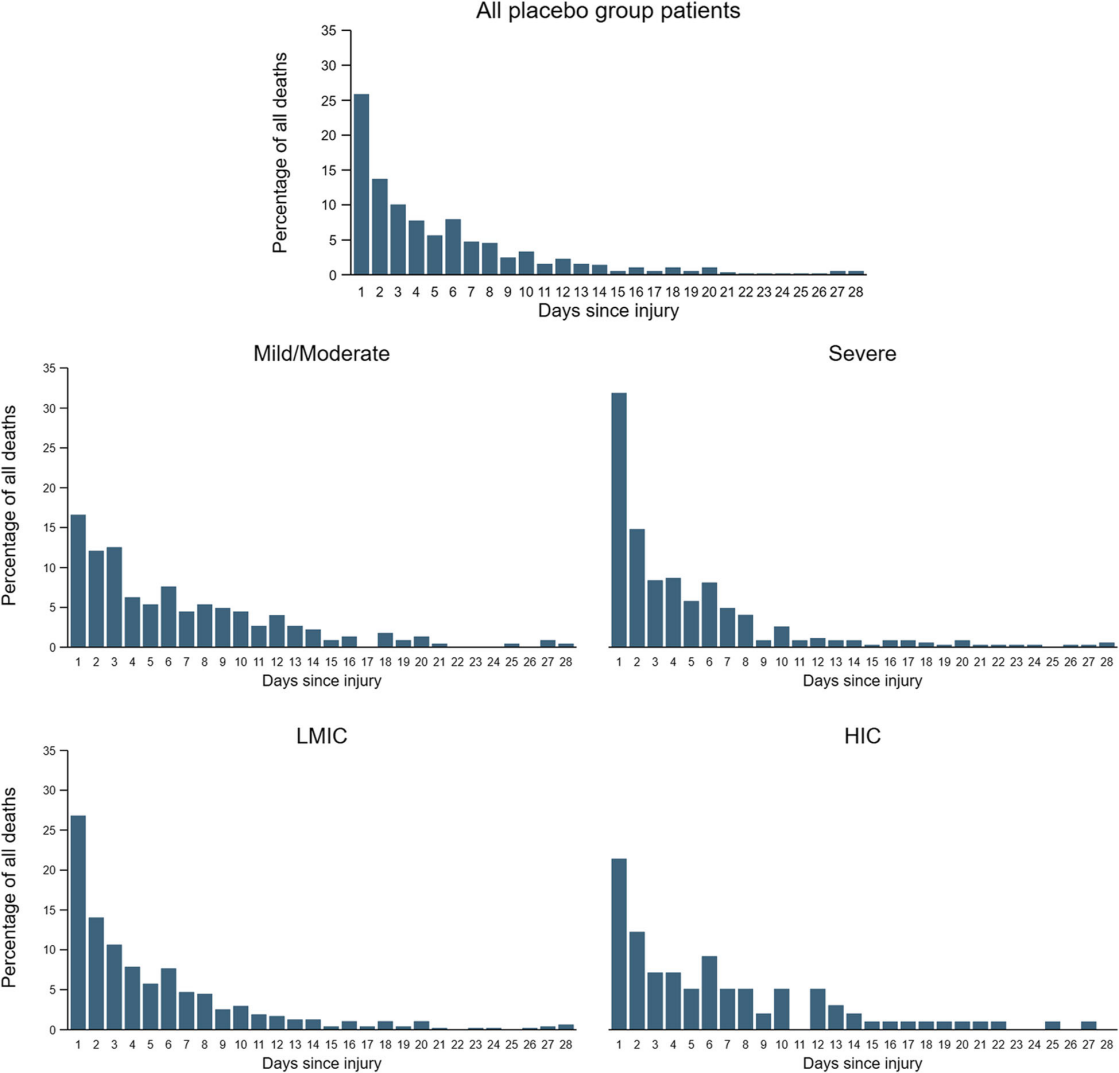
Days from injury to death in placebo group patients randomised within 3 h of injury overall and by severity (GCS) and country income, excluding those with GCS 3 or bilateral unreactive pupils

Among the 12,639 randomised patients with outcome data available, 9127 were treated within 3 h of injury. A total of 1490 patients had GCS score of 3 or bilateral unreactive pupils at baseline (16.3%), leaving 7637 patients for analysis. There were 1112 deaths from all causes within 28 days, of which 259 (23.3%) occurred within 24 h of injury (early deaths) and 853 (76.7%) were beyond 24 h of injury. Figure 1 shows the time interval from injury to death in placebo-treated patients overall and according to severity and country income. Overall, the proportion of early deaths was larger in severe head injury (28.1%) and in LMICs (24.1%).

### Effect of tranexamic acid on early deaths

The risk of early death was lower in patients with mild-to-moderate head injury compared to severe head injury (1.1% vs 9.9%) and in HICs compared to LMICs (2.0% vs 3.8%). The risk of early death was reduced with tranexamic acid (112 (2.9%) deaths in the tranexamic acid group vs 147 (3.9%) deaths in the placebo group; risk ratio [RR] RR 0.74, 95% CI 0.58–0.94; see Table 1). There was no evidence that the effect of tranexamic acid on early deaths varied by severity (heterogeneity *p* = 0.64) or by country income (heterogeneity *p* = 0.68). When 114 (1.5%) patients with hypotension (SBP < 90 mmHg) at baseline were excluded from the analyses, the results were essentially the same (106 (2.8%) deaths in the tranexamic acid group vs 143 (3.9%) deaths in the placebo group; RR 0.72, 95% CI 0.56–0.92). The effect of tranexamic acid on early death was smaller (261 vs 315 events; RR 0.81, 95% CI 0.69–0.95) when we included patients who had a GCS score of 3 or bilateral unreactive pupils at baseline (see Appendix Table 1). The effect was larger when the analysis was restricted to head injury-related deaths only (Appendix Tables 2 and 3).

**Table 1:** Effect of early tranexamic acid on all-cause mortality within 24 h of injury, after 24 h and at 28 days stratified by severity and country income level in patients randomised within 3 h of injury, excluding those with a GCS score of 3 or bilateral unreactive pupils

	Within 24 h			After 24 h			At 28 days		
	TXA	Placebo	RR (95% CI)	TXA	Placebo	RR (95% CI)	TXA	Placebo	RR (95% CI)
	*n* (%)	*n* (%)		*n* (%)	*n* (%)		*n* (%)	*n* (%)	
***All patients***	112 (2.9)	147 (3.9)	0.74 (0.58–0.94)	432 (11.5)	421 (11.7)	0.98 (0.69–1.12)	544 (14.0)	568 (15.1)	0.93 (0.83–1.03)
***Severity***									
**Mild/moderate**	25 (0.9)	37 (1.3)	0.66 (0.40–1.09)	163 (5.8)	186 (6.9)	0.85 (0.69–1.04)	188 (6.7)	223 (8.1)	0.82 (0.68–0.99)
**Severe**	87 (8.5)	110 (11.3)	0.75 (0.58–0.98)	269 (28.7)	235 (27.2)	1.05 (0.91–1.22)	356 (34.7)	345 (35.4)	0.98 (0.87–1.10)
***Country income***									
**LMIC**	98 (3.3)	126 (4.4)	0.75 (0.58–0.98)	363 (12.6)	344 (12.5)	1.01 (0.88–1.16)	461 (15.5)	470 (16.3)	0.95 (0.84–1.07)
**HIC**	14 (1.5)	21 (2.4)	0.65 (0.33–1.26)	69 (7.7)	77 (9.0)	0.86 (0.63–1.18)	83 (9.2)	98 (11.1)	0.82 (0.62–1.08)

### Effect of tranexamic acid on deaths after 24 h

The risk of death more than 24 h after injury was lower in patients with mild-to-moderate head injury compared to severe head injury (6.3% vs 25.2%) and in HICs compared to LMICs (8.2% vs 12.1%). The risk of death from all causes beyond 24 h of injury was similar in the tranexamic acid and placebo groups (432 (11.5%) deaths in the tranexamic acid group vs 421 (11.7%) deaths in the placebo group; RR 0.98, 95% CI 0.69–1.12; see Table 1). The effect on deaths beyond 24 h was similar by severity (heterogeneity *p* = 0.088) and country income (heterogeneity *p* = 0.36).

### Effect of tranexamic acid on deaths at 28 days

The risk of death at 28 days was lower in mild-to-moderate head injury compared to severe head injury (7.4% vs 35.1%) and in HICs compared to LMICs (10.1% vs 15.9%). The risk of death from any cause at 28 days was 14.0% in the tranexamic acid group versus 15.1% in the placebo group (544 vs 568 events; RR 0.93, 95% CI 0.83–1.03; see Table 1). The effect of tranexamic acid on all-cause mortality at 28 days was similar by severity (heterogeneity *p* = 0.11) and country income (heterogeneity *p* = 0.35).

### Effect of tranexamic acid on vascular occlusive events

Among the 12,639 randomised patients with outcome data, there were 203 (1.6%) fatal or non-fatal vascular occlusive events. The absolute risk of vascular occlusive events in all patients was lower in mild-to-moderate head injury than in severe head injury (1.2% vs 2.4%) and in LMICs compared to HICs (1.0% vs 3.0%). The risk of vascular occlusive events was 1.6% in both the tranexamic acid and placebo groups (101 vs 102 events; RR 0.98, 95% CI 0.74–1.28; see Table 2).

**Table 2:** Effect of tranexamic acid on vascular occlusive events (fatal and non-fatal) at 28 days in all patients, stratified by severity and country income level

	TXA			Placebo			RR (95% CI)
	*N*	*n*	(%)	*N*	*n*	(%)	
***All patients***	6359	101	(1.6)	6280	102	(1.6)	0.98 (0.74–1.28)
***Severity***							
**Mild/moderate**	4066	41	(1.0)	3997	52	(1.3)	0.76 (0.52–1.16)
**Severe**	2264	60	(2.7)	2247	50	(2.2)	1.19 (0.82–1.73)
***Country income***							
**LMIC**	4375	50	(1.1)	4330	35	(0.8)	1.41 (0.92–2.17)
**HIC**	1984	51	(2.6)	1950	67	(3.4)	0.75 (0.52–1.07)

### The results of the CRASH-3 trial in context

**Fig. 2:**
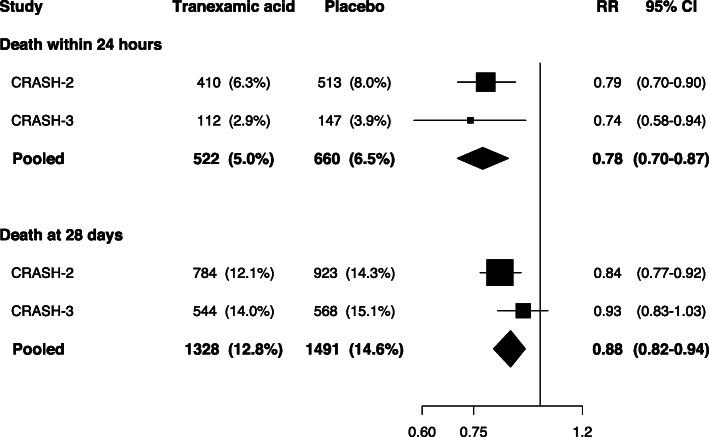
Evidence on the effect of early tranexamic acid on all-cause mortality within 24 h and 28 days of injury, excluding patients with a GCS score of 3 or bilateral unreactive pupils at baseline

When the CRASH-2 and CRASH-3 trial data were pooled in a one-stage individual patient data meta-analysis, early tranexamic acid reduced death within 24 h of injury (RR 0.78, 95% CI 0.70–0.87) and death within 28 days (RR 0.88, 95% CI 0.82–0.94), with no evidence of heterogeneity by trial (death within 24 h *p* = 0.60; death within 28 days *p* = 0.18; see Fig. 2). Adjusting for time to treatment made no difference to the results. For deaths with 24 h of injury, the adjusted RR = 0.78 (95% CI 0.70–0.87), and for death within 28 days the adjusted RR = 0.88 (95% CI 0.82–0.94). When a US trial of pre-hospital tranexamic acid for isolated TBI was included in an aggregate data meta-analysis on death from any cause at 28 days, the results were identical (RR 0.88, 95% CI 0.82–0.94), with no evidence of heterogeneity by trial (*p* = 0.41). There was no difference in the risk of vascular occlusive events between treatment groups (RR 0.87, 95% CI 0.74–1.02), again with no heterogeneity by trial (*p* = 0.42).

## Discussion

Based upon this post hoc, exploratory analysis of the CRASH-3 trial, tranexamic acid reduces deaths on the day of the injury regardless of TBI severity and country income but has no apparent effect on deaths beyond the day of the injury. The effect of tranexamic acid on all-cause mortality at 28 days is a weighted average of these early and late effects and, although diluted toward the null, is similar to the results of the CRASH-2 trial and indicative of a survival benefit.

Because a larger proportion of deaths in the CRASH-3 trial occurred after 24 h (69% in CRASH-3 versus 43% in CRASH-2), the effect on mortality at 28 days is smaller (more diluted) in the CRASH-3 trial, although there is no evidence of heterogeneity. As anticipated in the statistical analysis plan, the effect is smaller when including patients with un-survivable injuries prior to treatment. Tranexamic acid did not increase the risk of adverse vascular occlusive events in trauma patients.

Because our choice of head injury death as the primary outcome measure was criticised, these analyses report all-cause mortality. The trial inclusion criteria were clinical and reflect the situation that doctors face in practice. We enrolled TBI patients within 3 h of injury if they had no significant extra-cranial bleeding. The effect of tranexamic acid on early deaths is not explained by undiagnosed extra-cranial bleeding. Only 1.5% of patients had hypotension (SBP < 90 mmHg) at baseline and only 11 of the 1112 deaths (six in the tranexamic acid group and five in the placebo group) were classified as extracranial bleeding deaths. When patients with hypotension are excluded, the results are the same. The reduction in all-cause mortality within 24 h strongly suggests that tranexamic acid reduces intracranial bleeding deaths.

We conducted the CRASH-3 trial because there was reason to believe that tranexamic acid could reduce bleeding-related head injury deaths. Increased fibrinolysis is common in TBI patients and worsens intracranial bleeding. The CRASH-2 trial in 20,211 polytrauma patients (extra-cranial and intra-cranial injury) with significant bleeding found that tranexamic acid reduces mortality, primarily by reducing bleeding deaths on the day of the injury [15]. Because the CRASH-2 trial was large, this early benefit was still apparent at 28 days, although ‘diluted’ by non-bleeding deaths. The CRASH-3 trial was smaller than the CRASH-2 trial, and so despite the higher mortality rate, there were fewer deaths and less statistical power to detect the diluted effect on all-cause mortality at 28 days. A non-significant difference between two groups in a randomised trial can be real difference that is not significant due to a lack of power, or it can be a difference that has occurred by chance. In this case (Table 1), there is a large reduction in deaths within 24 h with tranexamic acid (RR = 0.74) that is highly statistically significant and consistent with the expected biological effects of tranexamic acid but no apparent reduction in deaths beyond 24 h (RR = 0.98). Because the relative risk at 28 days is a weighted average of these effects, the modest reduction in death at 28 days (RR = 0.93) is not statistically significant. We believe the reduction in deaths at 28 days is a real reduction that is not significant due to a lack of statistical power. This interpretation is consistent with biology (intracranial bleeding occurs early, and there is little or no tranexamic acid in the body beyond 24 h) and as shown in the next paragraph is mathematically consistent with dilution. The reduction in deaths at 28 days in the CRASH-3 trial is similar to that seen in the larger (and more powerful) CRASH-2 trial, and when the results are pooled, the reduction in deaths at 28 days with tranexamic acid is highly significant. However, we accept that can never rule out chance as a potential explanation.

**Fig. 3:**
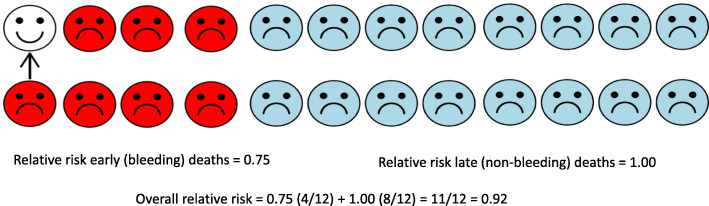
Hypothetical trial in which the effect on all-cause mortality is a weighted average of the effect on cause-specific mortality. The trial treatment reduces the risk of early (bleeding) deaths by one quarter (RR = 0.75) but has no effect on late (non-bleeding) deaths (RR = 1.00). The overall relative risk for all-cause mortality at the end of follow-up is a weighted average of these relative risks (RR = 0.92)

Because ‘dilution’ is key to understanding the CRASH-3 results, it is best considered quantitatively. Figure 3 shows results from a hypothetical trial in which the treatment reduces the risk of early bleeding deaths (red circles) by one quarter (relative risk = 0.75), but has no effect (relative risk = 1.00) on later non-bleeding deaths (blue circles). The relative risk at the end of the follow-up period is a weighted average of these relative risks: relative risk = 0.75(4/12) + 1.0(8/12) = 11/12 = 0.92, where (4/12) and (8/12) are the proportions of deaths in the untreated group that are early or late. Because the relative risk at the end of follow-up is closer to the null (0.92 versus 0.75), and smaller effects are harder to detect, the treatment effect is less visible and, in this sense, is diluted. But the biological effect did not change. It was not offset by any harm but was simply obscured by deaths unrelated to its mechanism of action. Deaths that are inevitable before randomisation also dilute treatment effects. Many patients with a GCS score of 3 or unreactive pupils have un-survivable injuries and will die soon after admission regardless of treatment. Errors in the estimation of the time of injury could result in the inclusion of patients outside the eligibility time window, and because late treatment is less effective, this will also cause dilution. This is most relevant in LMICs where patients are often taken to hospital by bystanders or family members in private vehicles with no recording of the time of injury.

Because no treatment has effects on all causes of death, all-cause mortality at 28 days is a composite outcome that combines deaths affected by the trial treatment with those that are unaffected by it [16]. Using all-cause mortality to assess the ‘true’ effect of a treatment has counterintuitive consequences since it means that the effect of any given treatment depends on the effects of all the others. An antibiotic that reduces pneumonia deaths in week 2, by reducing the proportion of late deaths, will appear to increase the effectiveness of a treatment for early bleeding. Because the proportion of late deaths varies with injury severity and in different locations, all-cause mortality is not generalisable. The only generalisable measure is the undiluted biological effect of the trial treatment.

## Conclusions

Tranexamic acid safely reduces early deaths in non-moribund TBI patients regardless of TBI severity or country income. The effect of tranexamic acid in patients with isolated TBI is similar to that in polytrauma, reducing deaths on the day of the injury by over 20% in the CRASH-2 and the CRASH-3 trials. Tranexamic acid is included in treatment guidelines for the pre-hospital care of bleeding trauma patients, but patients with isolated TBI were excluded. The CRASH-3 trial data support the reconsideration of tranexamic acid for administration in isolated TBI, and even severely injured patients appear to benefit when treated soon after injury.

## Supplementary information

Additional file 1: **Supplementary Table 1.** Effect of tranexamic acid on all-cause mortality within 24 hours of injury, after 24 hours and at 28 days stratified by severity and country income in patients randomised within 3 hours of injury. **Supplementary Table 2.** Effect of tranexamic acid on head injury death within 24 hours, after 24 hours and at 28 days by severity and country income in patients randomised within 3 hours of injury, excluding those with GCS 3 or bilateral unreactive pupils. **Supplementary Table 3.** Effect of tranexamic acid on head injury death within 24 hours, after 24 hours and at 28 days by severity and country income in patients randomised within 3 hours of injury.

## Data Availability

Following publication of the primary and secondary analyses, individual de-identified patient data from the CRASH-3 trial will be made available via our data sharing portal, The Free Bank of Injury and Emergency Research Data (freeBIRD) website (http://freebird.Lshtm.ac.uk) indefinitely. The CRASH-2 trial data is already available. The trial protocols, statistical analysis plans and trial publications will be freely available online. The trial protocol, statistical analysis plan and trial publications will be freely available at http://www.txacentral.org/.
