# Supplementary material for: Understanding the neuroprotective effect of tranexamic acid: an exploratory analysis of the CRASH-3 randomised trial
Source: Crit Care. 2020 Nov 11;24:560. doi: 10.1186/s13054-020-03243-4 (PMC7657351; doi:10.1186/s13054-020-03243-4)
Supplement: Supplementary file 1 — Supplementary Table 1. Effect of tranexamic acid on all-cause mortality within 24 hours of injury, after 24 hours and at 28 days stratified by severity and country income in patients randomised within 3 hours of injury. Supplementary Table 2. Effect of tranexamic acid on head injury death within 24 hours, after 24 hours and at 28 days by severity and country income in patients randomised within 3 hours of injury, excluding those with GCS 3 or bilateral unreactive pupils. Supplementary Table 3. Effect of tranexamic acid on head injury death within 24 hours, after 24 hours and at 28 days by severity and country income in patients randomised within 3 hours of injury. [file 13054_2020_3243_MOESM1_ESM.docx]

***Supplementary Table 1*. Effect of tranexamic acid on all-cause mortality within 24 hours of injury, after 24 hours and at 28 days stratified by severity and country income in patients randomised within 3 hours of injury**

|  | **Within 24 hours** | |  | **After 24 hours** | |  | **At 28 days** |  |  |
| --- | --- | --- | --- | --- | --- | --- | --- | --- | --- |
|  |  |  |  |  |  |  |  |  |  |
|  | **TXA** | **Placebo** | **RR (95% CI)** | **TXA** | **Placebo** | **RR (95% CI)** | **TXA** | **Placebo** | **RR (95% CI)** |
|  | n (%) | n (%) |  | n (%) | n (%) |  | n (%) | n (%) |  |
|  |  |  |  |  |  |  |  |  |  |
| ***All patients*** | 261 (5.7) | 315 (7.0) | 0.81 (0.69-0.95) | 669 (15.4) | 633 (15.1) | 1.02 (0.92-1.13) | 930 (20.2) | 948 (21.0) | 0.96 (0.89-1.04) |
|  |  |  |  |  |  |  |  |  |  |
| ***Severity*** |  |  |  |  |  |  |  |  |  |
| **Mild/moderate** | 26 (0.9) | 38 (1.4) | 0.67 (0.41-1.09) | 169 (6.0) | 192 (7.0) | 0.85 (0.70-1.04) | 195 (6.9) | 230 (8.3) | 0.82 (0.69-0.99) |
| **Severe** | 235 (13.5) | 277 (16.2) | 0.83 (0.71-0.98) | 500 (33.2) | 441 (30.8) | 1.08 (0.97-1.20) | 735 (42.3) | 718 (42.0) | 1.01 (0.93-1.09) |
|  |  |  |  |  |  |  |  |  |  |
| ***Country income*** |  |  |  |  |  |  |  |  |  |
| **LMIC** | 215 (6.5) | 252 (7.7) | 0.84 (0.70-1.00) | 499 (16.0) | 471 (15.6) | 1.03 (0.92-1.15) | 714 (21.4) | 723 (22.1) | 0.97 (0.89-1.06) |
| **HIC** | 46 (3.6) | 63 (5.1) | 0.70 (0.49-1.02) | 170 (13.8) | 162 (13.8) | 1.00 (0.92-1.13) | 216 (16.8) | 225 (18.2) | 0.93 (0.78-1.10) |

***Supplementary Table 2*. Effect of tranexamic acid on head injury death within 24 hours, after 24 hours and at 28 days by severity and country income in patients randomised within 3 hours of injury, excluding those with GCS 3 or bilateral unreactive pupils**

|  | **Within 24 hours** | |  | **After 24 hours** | |  | **At 28 days** |  |  |
| --- | --- | --- | --- | --- | --- | --- | --- | --- | --- |
|  |  |  |  |  |  |  |  |  |  |
|  | **TXA** | **Placebo** | **RR (95% CI)** | **TXA** | **Placebo** | **RR (95% CI)** | **TXA** | **Placebo** | **RR (95% CI)** |
|  | n (%) | n (%) |  | n (%) | n (%) |  | n (%) | n (%) |  |
|  |  |  |  |  |  |  |  |  |  |
| ***All patients*** | 105 (2.7) | 142 (3.8) | 0.72 (0.56-0.92) | 380 (10.1) | 383 (10.6) | 0.95 (0.83-1.09) | 485 (12.5) | 525 (14.0) | 0.89 (0.80-1.00) |
|  |  |  |  |  |  |  |  |  |  |
| ***Severity*** |  |  |  |  |  |  |  |  |  |
| **Mild/moderate** | 23 (0.8) | 35 (1.3) | 0.64 (0.38-1.08) | 137 (4.9) | 165 (6.1) | 0.80 (0.64-1.00) | 160 (5.7) | 200 (7.3) | 0.78 (0.64-0.95) |
| **Severe** | 82 (8.0) | 107 (11.0) | 0.73 (0.55-0.96) | 243 (25.9) | 218 (25.2) | 1.03 (0.88-1.20) | 325 (31.7) | 325 (33.4) | 0.95 (0.84-1.08) |
|  |  |  |  |  |  |  |  |  |  |
| ***Country income*** |  |  |  |  |  |  |  |  |  |
| **LMIC** | 94 (3.2) | 121 (4.2) | 0.75 (0.58-0.98) | 327 (11.4) | 322 (11.7) | 0.97 (0.84-1.12) | 421 (14.2) | 443 (15.4) | 0.92 (0.81-1.04) |
| **HIC** | 11 (1.2) | 21 (2.4) | 0.51 (0.25-1.05) | 53 (5.9) | 61 (7.1) | 0.84 (0.59-1.19) | 64 (7.1) | 82 (9.3) | 0.76 (0.55-1.04) |

***Supplementary Table 3*. Effect of tranexamic acid on head injury death within 24 hours, after 24 hours and at 28 days by severity and country income in patients randomised within 3 hours of injury**

|  | **Within 24 hours** | |  | **After 24 hours** | |  | **At 28 days** |  |  | |
| --- | --- | --- | --- | --- | --- | --- | --- | --- | --- | --- |
|  |  |  |  |  |  |  |  |  |  | |
|  | **TXA** | **Placebo** | **RR (95% CI)** | **TXA** | **Placebo** | **RR (95% CI)** | **TXA** | **Placebo** | **RR (95% CI)** |  |
|  | n (%) | n (%) |  | n (%) | n (%) |  | n (%) | n (%) |  |  |
|  |  |  |  |  |  |  |  |  |  |  |
| ***All patients*** | 253 (5.5) | 306 (6.8) | 0.81 (0.69-0.95) | 602 (13.8) | 586 (14.0) | 0.99 (0.89-1.10) | 855 (18.5) | 892 (19.8) | 0.94 (0.86-1.02) |  |
|  |  |  |  |  |  |  |  |  |  |  |
| ***Severity*** |  |  |  |  |  |  |  |  |  |  |
| **Mild/moderate** | 24 (0.8) | 36 (1.3) | 0.65 (0.39-1.08) | 142 (5.0) | 171 (6.3) | 0.80 (0.65-1.00) | 166 (5.8) | 207 (7.5) | 0.78 (0.64-0.95) |  |
| **Severe** | 229 (13.2) | 270 (15.8) | 0.83 (0.71-0.98) | 460 (30.6) | 415 (29.0) | 1.06 (0.94-1.18) | 689 (39.6) | 685 (40.1) | 0.99 (0.91-1.07) |  |
|  |  |  |  |  |  |  |  |  |  |  |
| ***Country income*** |  |  |  |  |  |  |  |  |  |  |
| **LMIC** | 210 (6.3) | 244 (7.4) | 0.85 (0.71-1.01) | 458 (14.7) | 447 (14.8) | 0.99 (0.88-1.12) | 668 (20.1) | 691 (21.1) | 0.95 (0.87-1.05) |  |
| **HIC** | 43 (3.4) | 62 (5.0) | 0.67 (0.46-0.98) | 144 (11.7) | 139 (11.8) | 0.98 (0.79-1.22) | 187 (14.6) | 201 (16.2) | 0.90 (0.75-1.08) |  |
